# Supplementary material for: Glycogen deficiency enhances carbon partitioning into glutamate for an alternative extracellular metabolic sink in cyanobacteria
Source: Commun Biol. 2024 Feb 26;7:233. doi: 10.1038/s42003-024-05929-9 (PMC10897207; doi:10.1038/s42003-024-05929-9)

Supplementary Figure 1. Levels of intracellularly accumulated metabolites during the ^{13}C-labeling experiment. The wild-type (blue circles) and Δ*glgC* mutant (orange squares) cyanobacteria were cultured using media with initial nitrate concentrations of 7.5 mM (low nitrogen, LN) and 17.6 mM (high nitrogen, HN) for 5 days. Subsequently, 25 mM $NaH^{13}CO_3$ was added to the culture medium as a carbon source. After 0 to 12 h cultivation, levels of intracellular metabolites (**a**) and the ratio of ^{13}C in the total carbon (^{13}C fraction, shown in Figure 3) were determined. Levels of ^{13}C-labeled metabolites (**b**) were calculated by multiplying the levels of intracellular metabolites with the ^{13}C fraction. 2-OG: 2-oxoglutarate, DCW: dry cell weight, Glu: glutamate, Gln: glutamine. Values are shown as the mean ± standard deviation of three replicate experiments (*P < 0.05 by Welch's t test).

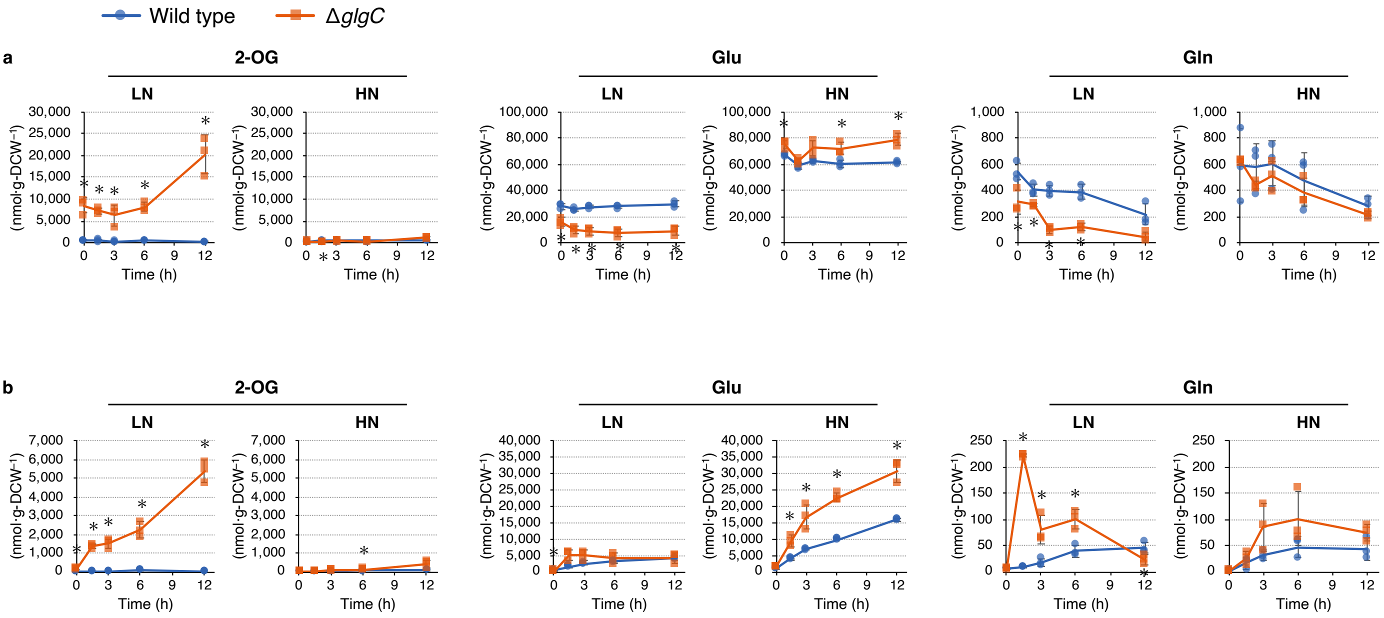

Supplement: Supplementary file 2 — Supplementary Fig. 1 [file 42003_2024_5929_MOESM2_ESM.pdf]
