# Supplementary material for: Glycogen deficiency enhances carbon partitioning into glutamate for an alternative extracellular metabolic sink in cyanobacteria
Source: Commun Biol. 2024 Feb 26;7:233. doi: 10.1038/s42003-024-05929-9 (PMC10897207; doi:10.1038/s42003-024-05929-9)
Supplement: Supplementary file 3 — Description of Additional Supplementary Files [file 42003_2024_5929_MOESM3_ESM.pdf]

Description of Additional Supplementary Files

File name: Supplementary Data 1
Description: The source data underlying Figures 1, 2, 3, and 4 in the paper.
